# Eating disorder mentor program

**DOI:** 10.1186/2050-2974-1-S1-O11

**Published:** 2013-11-14

**Authors:** Krystyn Smale, Jane Pitt

**Affiliations:** 1 Eating Disorders Unit, NorthWestern Mental Health, Royal Melbourne Hospital, Australia; 2 Eating Disorders Victoria

## 

Developing a sense of hope is a foundation for recovery. How can we inspire hope and motivation in those who are suffering from an eating disorder and the struggles that ensue?

The Eating Disorder Mentor Program is a collaborative project between The Eating Disorder service at Royal Melbourne Hospital (ED-RMH) and Eating Disorders Victoria (EDV).

It's an opportunity for people with a lived experience of an eating disorder to tell their recovery story and share their insight with current patients. Research has shown the many benefits of peer support which include increased feelings of hope for patients and the learning of practical strategies to assist their recovery. Mentors gain skill and satisfaction from seeing their lived experience used as a valuable source of expertise in promoting recovery.

This presentation will:

• Give an overview of the program including the aims, processes and results

• Explore the value achieved by collaboration between a community based mental health provider and a specialist tertiary health service

• Highlight the benefit of combining the experience of both health professionals and consumers

This abstract was presented in the **Care in Inpatient and Community Settings** stream of the 2013 ANZAED Conference.

